# Double-antiangiogenic protein DAAP targeting vascular endothelial growth factor A and angiopoietins attenuates collagen-induced arthritis

**DOI:** 10.1186/ar4265

**Published:** 2013-08-14

**Authors:** Young-Sool Hah, Young Jun Koh, Hye Song Lim, Hyun-Ok Kim, Yun-Hong Cheon, Hae Sook Noh, Kyu Yun Jang, Sang Yong Lee, Gyun Min Lee, Gou Young Koh, Sang-Il Lee

**Affiliations:** 1 Clinical Research Institute, Gyeongsang National University Hospital, 79 Gangnam-Ro, Jinju, 660-702, South Korea; 2 National Research Laboratory of Vascular Biology and Stem Cells and Graduate School of Biomedical Science and Engineering, Korea Advanced Institute of Science and Technology (KAIST), 373-1 Guseong-dong, Daejeon, 305-701, South Korea; 3 Department of Internal Medicine and Institute of Health Science, Gyeongsang National University School of Medicine, 79 Gangnam-Ro, Jinju, 660-702, South Korea; 4 Department of Biochemistry and Neurobiology and Institute of Health Sciences, Gyeongsang National University School of Medicine, 79 Gangnam-Ro, Jinju, 660-702, South Korea; 5 Department of Pathology, Medical School, Chonbuk National University, San 2-20, Geumam-dong, Jeonju, 561-756, South Korea; 6 Department of Radiology, Medical School, Chonbuk National University, San 2-20, Geumam-dong, Jeonju, 561-756, South Korea; 7 Department of Biological Sciences and Graduate School of Nanoscience and Technology, Korea Advanced Institute of Science and Technology (KAIST), 373-1 Guseong-dong, Daejeon, 305-701, South Korea

## Abstract

**Introduction:**

Angiogenesis plays a critical role in synovial inflammation and joint destruction in rheumatoid arthritis (RA). Vascular endothelial growth factor A (VEGF-A) and angiopoietins are two important mediators of synovial angiogenesis. We have previously developed a novel chimeric decoy receptor, namely, double-antiangiogenic protein (DAAP), which can both bind VEGF-A and angiopoietins and block their actions. This study was performed to evaluate the antiarthritic effect of DAAP and the combination effect with the tumor necrosis factor α (TNF-α) inhibitor in collagen-induced arthritis (CIA).

**Methods:**

Recombinant DAAP, VEGF-Trap, Tie2-Fc and dimeric Fc proteins were produced and purified from CHO cells in large-scale bioreactors. CIA was induced in DBA/1 mice with type II collagen. The preventive effect of DAAP was determined and compared with other decoy receptors such as VEGF-Trap or Tie2-Fc, which block VEGF-A or angiopoietins, respectively. The clinical, radiographic, pathologic and immunohistochemical analyses were performed in CIA mice. The levels of matrix metalloprotease 3 (MMP-3) and interleukin 1β (IL-1β) were quantified by enzyme-linked immunosorbent assay, and receptor activator of nuclear factor κB ligand (RANKL) mRNA levels were measured by polymerase chain reaction. Finally, we investigated the combination effects of DAAP with a low dose of TNF-α decoy receptor (etanercept 10 mg/kg).

**Results:**

On the basis of clinical and radiographic evaluation, DAAP had a much greater inhibitory effect than VEGF-Trap or Tie2-Fc on arthritis severity and bone destruction. These inhibitory effects were accompanied by significantly diminishing pathologic abnormalities, CD31-positive vasculature and synovial infiltration by F4/80-positive macrophages. The levels of MMP-3, IL-1β and RANKL were much lower in the DAAP-injected group than those of the control. Furthermore, DAAP showed a therapeutic effect and a combination effect with etanercept when injected after arthritis onset in established CIA.

**Conclusions:**

DAAP has not only potent prophylactic effects on both inflammation and bone destruction but also therapeutic effects, alone and in combination with a TNF-α inhibitor in CIA mice. These results suggest that DAAP could be used as an effective new therapeutic agent for RA.

## Introduction

Rheumatoid arthritis (RA) is the most common inflammatory arthritis and a major cause of disability due to joint destruction and permanent deformity [1]. Angiogenesis plays a critical role in RA by promoting inflammatory cell infiltration and the development of pannus, aggressive tumorlike fibrovascular granulation tissue, which eventually leads to extensive joint destruction [2,3]. Thus, the inhibition of angiogenesis, which has long been studied in the treatment of malignancies, is emerging as a potential therapeutic approach for RA [3,4].

Multiple mediators have been implicated in the process of angiogenesis [5,6]. Among them, vascular endothelial growth factor A (VEGF-A) and VEGF receptor (VEGFR) are the most intensively studied key regulators of angiogenesis in inflammation [7]. VEGF-A also contributes directly to joint destruction by stimulating osteoclasts through upregulation of receptor activators of nuclear factor κB (RANK) in endothelial cells [8]. Thus, numerous therapies have been developed that specifically target these molecules in RA [4]. However, despite some positive findings, recent clinical trials in cancer patients treated with VEGF-A inhibitors have revealed disadvantages such as insufficiency, resistance and toxicities [9-11].

Angiopoietins, including angiopoietin 1 (Ang-1), Ang-2 and Ang-3/Ang-4, are other important angiogenic factors. They interact with tyrosine kinase with immunoglobulin and epidermal growth factor homology domain 2 (Tie2) receptors [12]. Numerous studies have demonstrated that Ang-1, Ang-2 and Tie2 levels are increased in RA tissues and that blocking them inhibits angiogenesis as well as arthritis development and progression in collagen-induced arthritis (CIA) [13-19]. In particular, many reports have shown that Ang-2 is the major angiopoietin that enhances tumor angiogenesis, along with several other growth factors, such as VEGF-A [12,20,21]. Indeed, the upregulation of angiopoietins may be a major mechanism underlying the inadequate therapeutic effects of VEGF-A pathway blockage [9,22]. Therefore, the discovery of a new agent, which could simultaneously block both VEGF-A and angiopoietins, will be needed to more effectively suppress pathologic angiogenesis in cancer and RA.

We have developed a novel chimeric decoy receptor, double-antiangiogenic protein (DAAP), which can bind the VEGF-A, placenta growth factor (PIGF) and the angiopoietins and thereby simultaneously block their actions [22]. A previous report showed that DAAP was highly effective for suppressing tumor angiogenesis and metastasis in implanted and spontaneous solid tumors, as well as for reducing ascites formation and vascular leakage in an ovarian carcinoma model, compared with VEGF-Trap or Tie2-Fc, which block only VEGF or angiopoietin signaling, respectively [22]. However, the question whether DAAP might be useful in RA has remained unanswered to date. Thus, we examined whether DAAP might show higher potency than VEGF-Trap or Tie2-Fc and whether it might represent a beneficial combinatory effect when combined with TNF-α inhibitor in an experimental model of RA.

## Materials and methods

### Generation of recombinant proteins

Recombinant proteins, including DAAP, VEGF-Trap, Tie2-Fc and dimeric Fc (Fc), were produced as previously described [22]. Briefly, the genes for DAAP, VEGF-Trap, Tie2-Fc and Fc were inserted into pCMV-dhfr2, transfected into dhfr-deficient CHO cells (DG44), selected with G418 and amplified by sequential increases in methotrexate. The clones with the highest production of each recombinant protein were selected and further grown to obtain suitable amounts of protein. Recombinant proteins in supernatants were purified by column chromatography with protein A agarose resin (OncogeneScience/Wilex, Cambridge, MA, USA). After purification, recombinant proteins were quantified using the Bradford assay and confirmed by Coomassie blue staining after SDS-PAGE.

### Collagen-induced arthritis experiment

DBA/1 mice were purchased from The Jackson Laboratory (Bar Harbor, ME, USA), and male DBA/1 mice (seven to nine weeks old) were immunized with 150 μg of bovine collagen type II (Chondrex, Redmond, WA, USA) emulsified with an equal volume of Complete Freund's Adjuvant (Chondrex). The day of the first immunization was defined as day 0. The mice were then boosted with an equal amount of bovine collagen type II emulsified in Incomplete Freund's Adjuvant on day 21. For the preventive experiment, CIA mice were given intraperitoneal injections of phosphate-buffered saline (PBS), Fc, VEGF-Trap, Tie2-Fc or DAAP (25 mg/kg, twice weekly) from day 21 to day 40. For the therapeutic experiment, following the development of clinical arthritis (average arthritis score of 2) on day 26, CIA mice were randomized and given intraperitoneal injections of Fc, DAAP (25 mg/kg every two days), etanercept (10 mg/kg every two days) or DAAP + etanercept. Clinical arthritis scores were evaluated using a scale of 0 to 3 for each paw with a total possible score of 12. Ankle thickness was measured with a caliper placed across the ankle joint at the widest point. All experimental animals used in this study were maintained under the protocol approved by Gyeongsang National University Institutional Animal Care and Use Committee (GLA-080822-M0060).

### Radiological examination

Plain radiographs of the knees and feet were obtained using a mammographic imager as previously described [23]. The degree of joint destruction and bone erosion scored on a scale from 0 to 5, where 0 is no damage; 1 is minor bone destruction observed in one enlightened spot; 2 is moderate change, two to four with spots in one area; 3 is marked changes, two to four spots in more than one area; 4 is severe erosion afflicting the joints; and 5 is complete destruction of the joints.

### Histological and morphometric analyses

Fixed joint tissues were decalcified and embedded in paraffin. Sections (5 µm) were stained with hematoxylin and eosin and Safranin O. The joint sections were scored for changes in synovial inflammation, pannus formation, cartilage damage and bone erosion, all on a 0- to 3-point scale. CD31 and F4/80 were immunohistochemically detected using a rabbit polyclonal antibody against CD31 (Thermo Scientific, Waltham, MA, USA) or a rat monoclonal antibody directed against F4/80 (Santa Cruz Biotechnology, Santa Cruz, CA, USA). The number of F4/80^+ ^cells and CD31^+ ^blood vessels were counted in eight randomly selected high-power fields (×400) for each knee and ankle joint.

### Enzyme-linked immunosorbent assay

Ankle joints and serum were harvested at day 40 from the CIA mice. The frozen joint tissues were pulverized in liquid nitrogen, and total protein extracts from the ankle joints were isolated from individual homogenized joints in lysis buffer (mammalian protein extraction reagent; Pierce Biotechnology, Rockford, IL, USA) containing Protease Inhibitor Cocktail (Calbiochem/EMD Millipore, Billerica, MA, USA). IL-1β, MMP-3 and VEGF were measured using commercially available enzyme-linked immunosorbent assay kits according to the manufacturer's instructions (R&D Systems, Minneapolis, MN, USA).

### Reverse transcription polymerase chain reaction

Total RNA was isolated and transcribed into cDNA using the iScript cDNA Synthesis Kit (Bio-Rad Laboratories, Hercules, CA, USA) according to the manufacturer's protocol. After denaturing at 85°C for five minutes and then cooling to 4°C, cDNA was amplified using PCR. Two microliters of denatured cDNA were amplified in a 50-μl final volume containing 1 U of ExTaq DNA polymerase (TaKaRa, Tokyo, Japan), 0.4 μM concentrations of each primer, Taq polymerase buffer and 2 mM concentrations of each deoxyribonucleotide triphosphate. PCR was performed in a thermal cycler (Bio-Rad Laboratories) using a program of 35 cycles at 95°C for 30 s, 56°C for 40 s and 72°C for 50 s with a final seven-minute extension at 72°C. The sense primer for RANKL was 5'-CCAGCATCAAAATCCCAAGTT-3', and the antisense primer was 5'-TCAAGGTTCTCAGTGGCACAT-3'. The sense primer for glyceraldehyde 3-phosphate dehydrogenase was 5'-AATGCATCCTGCACCACCAA-3', and the antisense primer was 5'-GTAGCCATATTCATTGTCAT-3'. The amplified products were subjected to electrophoresis on 1.2% agarose gel, and the results were visualized with ethidium bromide staining.

### Western blot analysis

Lysates were generated from ankle tissue in a radioimmunoprecipitation assay lysis buffer (20 mM Tris, pH 7.5, 140 mM NaCl, 1 mM ethylenediaminetetraacetic acid, 1% (vol/vol) Nonidet P-40, Protease Inhibitor Cocktail). The homogenated tissue was centrifuged at 12,000 *g *for 20 min at 4°C to remove insoluble debris. Equal amounts of the protein lysate were resolved on reducing SDS polyacrylamide gels and then electrophoretically transferred onto a nitrocellulose membrane (Amersham Pharmacia Biotech, Little Chalfont, UK). After blocking for one hour with 5% skimmed milk in a TBST buffer solution (10 mM Tris, 150 mM NaCl and 0.1% Tween 20), the membrane was incubated with primary antibodies against Ang-1 and Ang-2 (LifeSpan Biosciences, Seattle, WA, USA), followed by incubation with horseradish peroxidase-conjugated anti-rabbit immunoglobulin G (IgG) (Santa Cruz Biotechnology). The membrane was developed by SuperSignal West Pico Chemiluminescent Substrate (Pierce Biotechnology) and exposed to an X-ray film.

### Statistics

Values are expressed as the mean ± SE. Fisher's exact test was used to analyze the differences in incidence among groups. The Mann-Whitney *U *test was used to analyze arthritic severity and the radiographic and histologic findings. An unpaired Student's *t*-test and one-way analysis of variance were used to assess the other results. *P *≤ 0.05 was considered significant.

## Results

### Double-antiangiogenic protein exerts protective effects against inflammation and bone destruction

**Figure 1:**
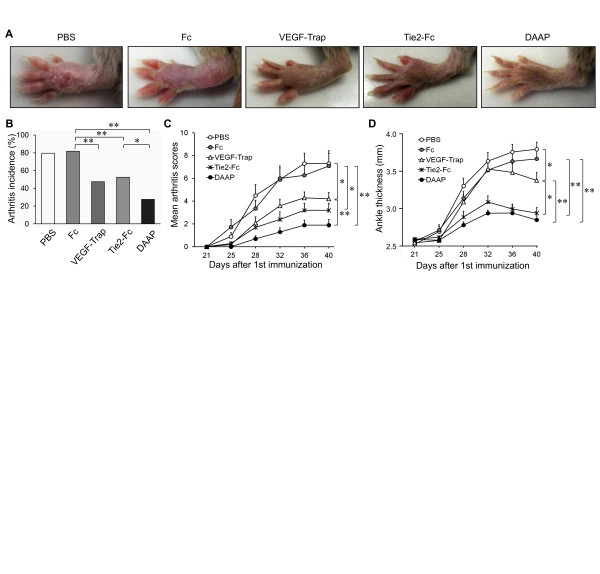
**Preventive effect of double-antiangiogenic protein on collagen-induced arthritis**. **(A) **Representative photographs showing the gross features of mice hind paws at day 40. Collagen-induced arthritis (CIA) mice were given intraperitoneal injections of phosphate-buffered saline (PBS), dimeric Fc (Fc), vascular endothelial growth factor (VEGF)-Trap, tyrosine kinase with immunoglobulin and epidermal growth factor homology domain 2 (Tie2)-Fc or double-antiangiogenic protein (DAAP) (25 mg/kg twice weekly, *n *= 10 for each group) from days 21 to 40. **(B) **Incidence of arthritis. **(C) **Arthritis scores. **(D) **Ankle thickness. Values are mean ± SE, *n *= 10 mice for each group. *P < 0.05, **P < 0.01.

**Figure 2:**
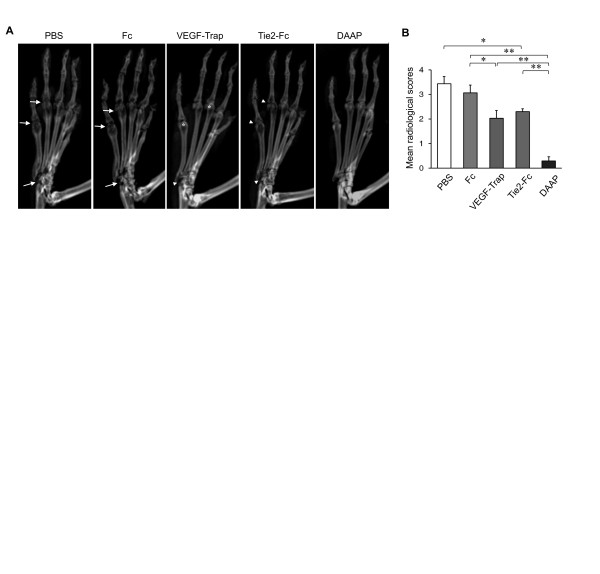
**Inhibitory effect of double-antiangiogenic protein on bone destruction in collagen-induced arthritis**. **(A) **Representative radiographs of hind paws. Note the severe bone erosions and joint damage (arrows), mild to moderate erosions (arrowheads) and periarticular osteopenia (asterisks). **(B) **Quantitative analysis of bone destruction. DAAP: Double-antiangiogenic protein; PBS: Phosphate-buffered saline; PCR: polymerase chain reaction; Tie2: Tyrosine kinase with immunoglobulin and epidermal growth factor homology domain 2; VEGF: Vascular endothelial growth factor. Values are mean ± SE, *n *= 20 for each group. *P < 0.05, **P < 0.01.

The severity of CIA, such as cumulative incidence, a clinical arthritis score and ankle thickness, indicated that arthritis was significantly attenuated with VEGF-Trap, Tie2-Fc and DAAP treatments compared with PBS or control Fc (Figures 1A through 1D). Particularly, the antiarthritic effect of DAAP was significantly greater than that of VEGF-Trap (*P *< 0.01) (Figures 1C and 1D). Severe bone erosion and joint damage were observed in PBS- or Fc-injected mice (arrows), but mild to moderate erosion (arrowheads) and periarticular osteopenia (asterisks) were observed in the mice injected with VEGF-Trap or Tie2-Fc (Figure 2A). Notably, DAAP-treated CIA mice showed minimal bone destruction and markedly decreased radiological scores compared with mice treated with VEGF-Trap or Tie2-Fc (*P *< 0.01) (Figure 2B). There was no difference in the suppressive effect on bone destruction between the VEGF-Trap- and Tie2-Fc-treated groups, although Tie2-Fc had a more potent inhibitory effect on inflammatory aspects, such as joint swelling and erythema. In comparison, DAAP showed potent protective effects against both inflammation and bone destruction.

**Figure 3:**
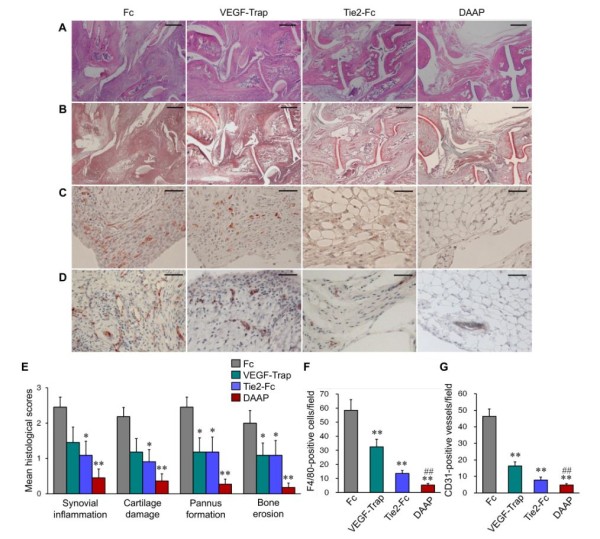
**Double-antiangiogenic protein reduces pathologic findings, infiltration of F4/80^+ ^macrophages and vessel formation in collagen-induced arthritis**. **(A) **and **(B) **Representative sections of ankle joints stained with hematoxylin and eosin and Safranin O, respectively (bars = 500 μm). **(C) **and **(D) **Representative images of synovium stained with F4/80 and CD31 (bars = 50 μm). **(E) **Quantitative scores of pathological abnormalities. **(F) **Mean number of infiltrating F4/80**^+ ^**macrophages. **(G) **Mean number of CD31**^+ ^**blood vessels in the synovium. DAAP: Double-antiangiogenic protein; Tie2: Tyrosine kinase with immunoglobulin and epidermal growth factor homology domain 2; VEGF: Vascular endothelial growth factor. The joint tissues were sampled at day 40. Values are mean ± SE, *n *= 18 to 20 for each group. *P < 0.05, **P < 0.01 vs. Fc; ^#^*P *< 0.05, ^##^*P *< 0.01 vs. VEGF-Trap.

The joints of Fc-injected CIA mice exhibited prominent synovial inflammation, cartilage damage, pannus formation and bone erosion. In contrast, VEGF-Trap- or Tie2-Fc-treated mice showed decreased pathological manifestations, and DAAP-treated mice exhibited dramatically reduced pathological abnormalities (Figures 3A and 3B). The histological scores for all parameters also showed decreased pathological abnormalities in the VEGF-Trap- or Tie2-Fc-treated groups and substantially fewer abnormalities in the DAAP-treated group (Figure 3E). Consistent with the results of our radiological findings, there was no difference in the suppressive effect on bone erosion between the VEGF-Trap and Tie2-Fc groups, although the Tie2-Fc group displayed less synovial inflammation than the VEGF-Trap group (Figure 3E).

### Double-antiangiogenic protein acts primarily by inhibiting blood vessel formation

Treatment with DAAP, VEGF-Trap or Tie2-Fc resulted in a decrease in CD31^+ ^blood vessels and F4/80^+ ^macrophages in the joint synovium compared with Fc (Figures 3C, D, F and 3G). Notably, the inhibitory effect of DAAP on blood vessel formation and macrophage infiltration was significantly greater than that of VEGF-Trap (*P *< 0.01) (Figures 3F and 3G).

**Figure 4:**
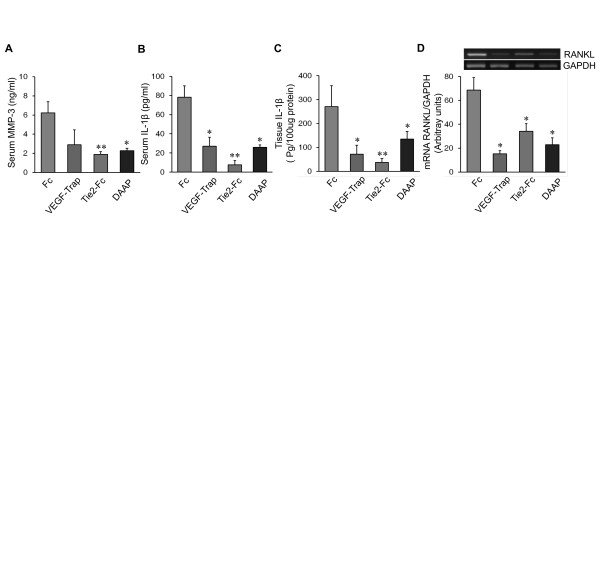
**Effects of double-antiangiogenic protein on the production of matrix metalloprotease 3, interleukin 1β and receptor activator of nuclear factor-κB ligand in collagen-induced arthritis**. **(A) **through **(C) **Comparative concentrations of serum matrix metalloprotease 3 (MMP-3) and interleukin 1β (IL-1β), and the IL-1β of tissue extracts from the hind paws of collagen-induced arthritis (CIA) mice injected with Fc, vascular endothelial growth factor (VEGF)-Trap, tyrosine kinase with immunoglobulin and epidermal growth factor homology domain 2 (Tie2)-Fc or double-antiangiogenic protein (DAAP). **(D) **Receptor activator of nuclear factor-κB ligand (RANKL) mRNA expression in the synovium of knee joints. A representative gel image is shown (upper) together with a graph comparing the semiquantitative polymerase chain reaction (PCR) data (lower). Values are presented as the mean relative density of the RANKL PCR product bands normalized to the glyceraldehyde 3-phosphate dehydrogenase (GAPDH) PCR product bands. In all experiments, *n *= 10 for each group. Values are mean ± SE. *P <0.05, **P <0.01 vs. Fc.

MMP-3 and IL-1β levels were decreased in mice injected with DAAP, VEGF-Trap and Tie2-Fc (Figures 4A through 4C). The levels of both MMP-3 and IL-1β in the serum and joint tissue of Tie2-Fc-treated CIA mice were considerably lower than in DAAP- or VEGF-Trap-treated mice. The RANKL expression of VEGF-Trap-treated CIA mice was significantly lower than in mice treated with DAAP or Tie2-Fc (Figure 4D). Taken together, these findings demonstrate that DAAP suppresses synovial inflammation, cartilage damage and bone destruction, primarily by inhibiting angiogenesis rather than by directly decreasing IL-1β, MMP-3 and RANKL.

### Double-antiangiogenic protein shows therapeutic and combination effects with etanercept in collagen-induced arthritis

**Figure 5:**
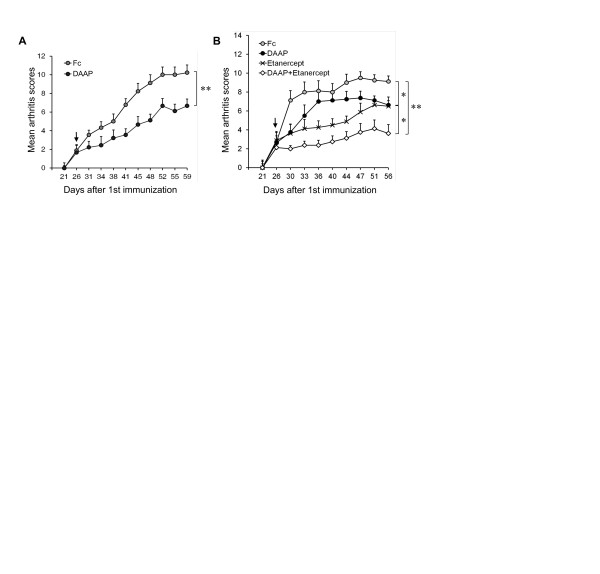
**Therapeutic and combination effects of double-antiangiogenic protein in collagen-induced arthritis**. **(A) **Therapeutic effect of double-antiangiogenic protein (DAAP). **(B) **Therapeutic effect of the combination of DAAP with etanercept. Following the development of clinical arthritis (average visual arthritis score of 2) on day 26 (arrow), collagen-induced arthritis mice were randomized to receive intraperitoneal injections of Fc, DAAP (25 mg/kg each every two days), etanercept (10 mg/kg every two days) or DAAP + etanercept (*n *= 8 or 9 mice for each group). Values are mean ± SE. *P < 0.05, **P < 0.01 vs. Fc.

To further test the possible therapeutic effect of DAAP, CIA mice that had begun developing arthritis were treated with either DAAP or Fc. DAAP-treated mice showed significantly reduced progression of arthritis compared to Fc-treated mice (Figure 5A). Additionally, combinatory effects between DAAP and a relatively low dose of etanercept (10 mg/kg) were tested and subsequently evaluated. The effect of DAAP combined with etanercept was significantly greater than that of either treatment alone on arthritic progression in mice with established CIA (Figure 5B).

### VEGF-A, Ang-1 and Ang-2 show different temporal patterns of expression during the course of collagen-induced arthritis

**Figure 6:**
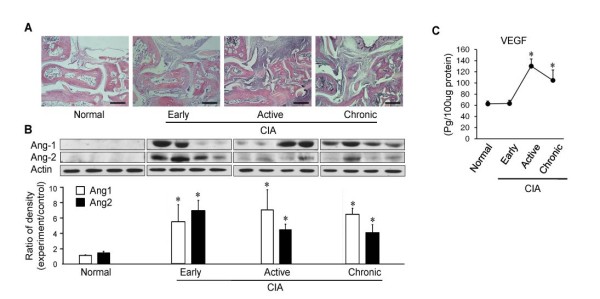
**Vascular endothelial growth factor A, angiopoietin 1 and angiopoietin 2 show different temporal patterns of expression during the course of collagen-induced arthritis**. Collagen-induced arthritis (CIA) was induced, and joint extracts were isolated from normal DBA1 mice and from early (day 26), active (day 38) and chronic (day 52) CIA mice (*n *= 4 for each group). **(A) **Representative sections of the ankle joints stained with hematoxylin and eosin (bars = 500 μm) from normal DBA1 and early, active and chronic CIA mice. **(B) **Sequential changes in the expression levels of angiopoietin 1 (Ang1) and Ang2 proteins in the joint tissue were determined by Western blot analysis. **(C) **The vascular endothelial growth factor A (VEGF-A) concentration in the joint tissue was determined by enzyme-linked immunosorbent assays. Values are mean ± SE. *P < 0.05 vs. normal.

These findings led us to further investigate why DAAP is more effective than VEGF-Trap or Tie2-Fc alone in the CIA model. Thus, joint extracts were isolated from normal DBA1 and CIA mice in the early (day 26), active (day 38) and chronic (day 52) phases and analyzed for temporal patterns of expression of VEGF-A, Ang-1 and Ang-2. In the early phase, Ang-1 and Ang-2 were increased. In contrast, VEGF-A was not increased. In the active phase, VEGF-A rose abruptly, Ang-1 continued to increase and Ang-2 decreased. In the chronic phase, Ang-1 was persistently high despite decreased VEGF-A (Figure 6). These results showed that the most important angiogenic growth factors vary according to the stage of CIA.

## Discussion

In this study, we found that DAAP produces better protective effects on both inflammation and bone destruction in CIA mice when compared with VEGF-Trap or Tie2-Fc. These effects of DAAP were correlated with significantly diminished pathologic abnormalities, CD31^+ ^vasculature and synovial infiltration by F4/80^+ ^macrophages, suggesting that DAAP acts primarily by inhibiting angiogenesis. We further demonstrated the therapeutic effect of DAAP and the beneficial combinatory effect of DAAP with TNF-α inhibitor in the established CIA mice. These data provide evidence for the usefulness of DAAP, which simultaneously blocks VEGF-A and angiopoietins as an effective new therapeutic agent for RA.

DAAP treatment showed more pronounced suppressive effects on arthritis severity than VEGF-Trap or Tie2-Fc alone in CIA mice. We propose the following explanations for why and how DAAP is more effective than VEGF-Trap or Tie2-Fc. First, arthritic synovium has strong expression and activation levels of VEGF-A, PIGF, Ang-1 and Ang-2, and their expression patterns were different according to disease stages of RA in our data and other reports [15,24-27]. Our previous study showed that DAAP is able to bind to VEGF-A, PIGF, Ang-1, Ang-2 and Ang-3/Ang-4. In comparison, VEGF-Trap was able to bind VEGF-A and PIGF but not any of the angiopoietins, whereas Tie2-Fc was able to bind all angiopoietins tested, but not VEGF-A and PIGF [22]. Second, the upregulation of Ang-2 is part of an angiogenic rescue when only VEGF-A/VEGFR2 signaling is blocked in tumor models [9,22]. Third, DAAP has a longer half-life than VEGF-Trap and Tie2-Fc, and the binding of either VEGF-A or Ang-2 to DAAP enhances additional binding of Ang-2 or VEGF-A to DAAP. Thus, DAAP appears to distribute well in the arthritic environment and to block VEGF-A and Ang-2 in a synergistic manner [22]. Taken together, the simultaneous blockage of VEGF and angiopoietin signaling by DAAP appears more effective in suppressing arthritis-associated angiogenesis than blockage by VEGF-Trap or Tie2-Fc separately.

Apart from its important function as a VEGF, angiopoietin/Tie-2 signaling induces the proliferation of RA synovial fibroblasts and promotes the proinflammatory activation of macrophages [16,17]. Especially, angiopoietin/Tie-2 signaling is an important mediator that links the proinflammatory TNF-α to pathologic angiogenesis [19,28]. VEGF-A aggravates joint destruction by directly increasing the differentiation and activity of osteoclasts [8,29]. In contrast, Ang1 can suppress osteoclast activity indirectly via enhanced osteoblast maturation [30,31]. In this study, MMP-3 and IL-1 levels were lower in the Tie2-Fc-treated group than DAAP or VEGF-Trap, but RANKL levels were lower in the VEGF-Trap-treated group than in the DAAP or Tie2-Fc groups. Collectively, these findings suggest that Tie2-Fc is more advantageous for suppressing inflammation than VEGF-Trap and that VEGF-Trap is more advantageous for preventing bone destruction than Tie2-Fc. DAAP is a chimeric decoy receptor that can block both the VEGF/VEGF-R and angiopoietin/Tie-2 pathways, simultaneously. Thus, DAAP has suppressive effects on both inflammation and bone destruction in CIA.

Numerous efforts to create a promising strategy for treating cancer and inflammatory arthritis have focused on disrupting the VEGF-A pathway [4,6]. Despite promising results, recent clinical trials with cancer patients treated with VEGF-A inhibitors have generated two puzzling questions. First, the anti-VEGF-A strategy is insufficient and evokes resistance. These findings reflect insufficient improvements in clinical outcomes, including benefits limited to short-term survival and the development of adaptive resistance, intrinsic nonresponsiveness and the rapid regrowth of tumor vessels after cessation of therapy [6,32]. In this regard, emerging evidence indicates that upregulation of angiopoietins may be a major mechanism underlying the inadequate therapeutic effects of VEGF-A pathway blockage [9,22]. Second, a number of adverse effects have been reported with anti-VEGF-A therapy, and some of these toxicities can be explained by the use of high doses of anti-VEGF-A [10,22]. Even though we administered a lower molar dosage of DAAP than VEGF-Trap or Tie2-Fc, DAAP displayed more pronounced effects. Therefore, DAAP may show greater antiarthritic activity and less toxicity than VEGF-Trap or Tie2-Fc in RA treatment.

Etanercept is a soluble TNF-α type II receptor linked to an IgG1-Fc moiety that binds to and inactivates TNF-α [33]. Although studies have demonstrated its efficacy in reducing inflammation and joint destruction, over 30% to 40% of anti-TNF-α-treated patients fail to achieve a response, and efficacy is lost in many patients during therapy [34,35]. Other shortcomings of anti-TNF-α therapies include increased rates of infection and malignancy [34]. In contrast, DAAP may be suitable to effectively treat both tumors and arthritis with no increased risk of infection. Our results showed the possible role of the combination of DAAP and TNF-α inhibitors in slowing arthritic progression more than either treatment alone. Therefore, DAAP may be used as a synergistic as well as an alternative treatment for RA patients who are responding poorly to a TNF-α blocking agent and for those patients at high risk of infection or malignancy.

This study has some limitations. Our previous study showed that DAAP is superior to the combined therapy of VEGF-Trap plus Tie2-Fc in suppressing tumor growth, angiogenesis and metastasis [22]. Those findings suggest that DAAP is also superior to the combined therapy of VEGF-Trap plus Tie2-Fc in CIA mice. However, we did not conduct CIA experiments to compare DAAP and the combined therapy of VEGF-Trap plus Tie2-Fc. Additionally, we proved only the priority of DAAP to be more effective than VEGF-Trap or Tie2-Fc in the prevention of CIA, not in the therapeutic effect in established CIA. Thus, further study is needed to prove these effects.

## Conclusions

We found that DAAP induces better protective effects than VEGF-Trap or Tie2-Fc on both inflammation and bone destruction in CIA mice. Additionally, we proved that DAAP has therapeutic effects and a beneficial combination effect with the TNF-α inhibitor on CIA. Our results raise the possibility that the coordinated blocking of angiogenic activities by DAAP might represent a promising and powerful strategy for the treatment of RA.

## Abbreviations

CIA: Collagen-induced arthritis; DAAP: Double-antiangiogenic protein; GAPDH: Glyceraldehyde 3-phosphate dehydrogenase; IL: Interleukin; MMP: Matrix metalloprotease; PCR: Polymerase chain reaction; RANKL: Receptor activator of nuclear factor-κB ligand; RA: Rheumatoid arthritis; TNF-α: Tumor necrosis factor α; Tie2: Tyrosine kinase with immunoglobulin and epidermal growth factor homology domain 2; VEGF: Vascular endothelial growth factor; VEGFR: Vascular endothelial growth factor receptor

## Competing interests

The authors declare that they have no competing interests.

## Authors' contributions

YSH, YJK, GYK and SIL designed, organized and performed the experiments, analyzed the data, generated the figures, and wrote the manuscript. HSL, HOK and YHC performed the CIA experiments. YJK and GML generated the recombinant proteins. HSN, KYJ and SYL performed the radiological and histological analyses. All authors read and approved the final version of the manuscript.

## References

[B1] Firestein GS Evolving concepts of rheumatoid arthritis Nature 2003 15 356 361 10.1038/nature01661 12748655

[B2] Szekanecz Z Koch AE Mechanisms of disease: angiogenesis in inflammatory diseases Nat Clin Pract Rheumatol 2007 15 635 643 10.1038/ncprheum0647 17968334

[B3] Firestein GS Starving the synovium: angiogenesis and inflammation in rheumatoid arthritis J Clin Invest 1999 15 3 4 10.1172/JCI5929 9884327 PMC407872

[B4] Lainer-Carr D Brahn E Angiogenesis inhibition as a therapeutic approach for inflammatory synovitis Nat Clin Pract Rheumatol 2007 15 434 442 17664950 10.1038/ncprheum0559

[B5] Ferrara N Kerbel RS Angiogenesis as a therapeutic target Nature 2005 15 967 974 10.1038/nature04483 16355214

[B6] Folkman J Angiogenesis: an organizing principle for drug discovery? Nat Rev Drug Discov 2007 15 273 286 10.1038/nrd2115 17396134

[B7] Olsson AK Dimberg A Kreuger J Claesson-Welsh L VEGF receptor signalling in control of vascular function Nat Rev Mol Cell Biol 2006 15 359 371 10.1038/nrm1911 16633338

[B8] Min JK Kim YM Kim YM Kim EC Gho YS Kang IJ Lee SY Kong YY Kwon YG Vascular endothelial growth factor up-regulates expression of receptor activator of NF-κB (RANK) in endothelial cells: concomitant increase of angiogenic responses to rank ligand J Biol Chem 2003 15 39548 39557 10.1074/jbc.M300539200 12893832

[B9] Loges S Mazzone M Hohensinner P Carmeliet P Silencing or fueling metastasis with VEGF inhibitors: antiangiogenesis revisited Cancer Cell 2009 15 167 170 10.1016/j.ccr.2009.02.007 19249675

[B10] Eskens FA Verweij J The clinical toxicity profile of vascular endothelial growth factor (VEGF) and vascular endothelial growth factor receptor (VEGFR) targeting angiogenesis inhibitors: a review Eur J Cancer 2006 15 3127 3139 10.1016/j.ejca.2006.09.015 17098419

[B11] Mancuso MR Davis R Norberg SM O'Brien S Sennino B Nakahara T Yao VJ Inai T Brooks P Freimark B Shalinsky DR Hu-Lowe DD McDonald DM Rapid vascular regrowth in tumors after reversal of VEGF inhibition J Clin Invest 2006 15 2610 2621 10.1172/JCI24612 17016557 PMC1578604

[B12] Augustin HG Koh GY Thurston G Alitalo K Control of vascular morphogenesis and homeostasis through the angiopoietin-Tie system Nat Rev Mol Cell Biol 2009 15 165 177 10.1038/nrm2639 19234476

[B13] Chen Y Donnelly E Kobayashi H Debusk LM Lin PC Gene therapy targeting the Tie2 function ameliorates collagen-induced arthritis and protects against bone destruction Arthritis Rheum 2005 15 1585 1594 10.1002/art.21016 15880817

[B14] Gravallese EM Pettit AR Lee R Madore R Manning C Tsay A Gaspar J Goldring MB Goldring SR Oettgen P Angiopoietin-1 is expressed in the synovium of patients with rheumatoid arthritis and is induced by tumour necrosis factor α Ann Rheum Dis 2003 15 100 107 10.1136/ard.62.2.100 12525377 PMC1754433

[B15] Raatz Y Ibrahim S Feldmann M Paleolog EM Gene expression profiling and functional analysis of angiogenic markers in murine collagen-induced arthritis Arthritis Res Ther 2012 15 R169 10.1186/ar3922 22817681 PMC3580563

[B16] Hashiramoto A Sakai C Yoshida K Tsumiyama K Miura Y Shiozawa K Nose M Komai K Shiozawa S Angiopoietin 1 directly induces destruction of the rheumatoid joint by cooperative, but independent, signaling via ERK/MAPK and phosphatidylinositol 3-kinase/Akt Arthritis Rheum 2007 15 2170 2179 10.1002/art.22727 17599743

[B17] Krausz S Garcia S Ambarus CA de Launay D Foster M Naiman B Iverson W Connor JR Sleeman MA Coyle AJ Hamann J Baeten D Tak PP Reedquist KA Angiopoietin-2 promotes inflammatory activation of human macrophages and is essential for murine experimental arthritis Ann Rheum Dis 2012 15 1402 1410 10.1136/annrheumdis-2011-200718 22730375

[B18] Jin P Zhang J Sumariwalla PF Ni I Jorgensen B Crawford D Phillips S Feldmann M Shepard HM Paleolog EM Novel splice variants derived from the receptor tyrosine kinase superfamily are potential therapeutics for rheumatoid arthritis Arthritis Res Ther 2008 15 R73 10.1186/ar2447 18593464 PMC2575619

[B19] DeBusk LM Chen Y Nishishita T Chen J Thomas JW Lin PC Tie2 receptor tyrosine kinase, a major mediator of tumor necrosis factor α-induced angiogenesis in rheumatoid arthritis Arthritis Rheum 2003 15 2461 2471 10.1002/art.11213 13130465

[B20] Hashizume H Falcón BL Kuroda T Baluk P Coxon A Yu D Bready JV Oliner JD McDonald DM Complementary actions of inhibitors of angiopoietin-2 and VEGF on tumor angiogenesis and growth Cancer Res 2010 15 2213 2223 10.1158/0008-5472.CAN-09-1977 20197469 PMC2840050

[B21] Oliner J Min H Leal J Yu D Rao S You E Tang X Kim H Meyer S Han SJ Hawkins N Rosenfeld R Davy E Graham K Jacobsen F Stevenson S Ho J Chen Q Hartmann T Michaels M Kelley M Li L Sitney K Martin F Sun JR Zhang N Lu J Estrada J Kumar R Coxon A Kaufman S Pretorius J Scully S Cattley R Payton M Coats S Nguyen L Desilva B Ndifor A Hayward I Radinsky R Boone T Kendall R Suppression of angiogenesis and tumor growth by selective inhibition of angiopoietin-2 Cancer Cell 2004 15 507 516 10.1016/j.ccr.2004.09.030 15542434

[B22] Koh YJ Kim HZ Hwang SI Lee JE Oh N Jung K Kim M Kim KE Kim H Lim NK Jeon CJ Lee GM Jeon BH Nam DH Sung HK Nagy A Yoo OJ Koh GY Double antiangiogenic protein, DAAP, targeting VEGF-A and angiopoietins in tumor angiogenesis, metastasis, and vascular leakage Cancer Cell 2010 15 171 184 10.1016/j.ccr.2010.07.001 20708158

[B23] Hah YS Lee YR Jun JS Lim HS Kim HO Jeong YG Hur GM Lee SY Chung MJ Park JW Lee SI Park BH A20 suppresses inflammatory responses and bone destruction in fibroblast-like synoviocytes and collagen-induced arthritic mice Arthritis Rheum 2010 15 2313 2321 10.1002/art.27545 20506221

[B24] Lu J Kasama T Kobayashi K Yoda Y Shiozawa F Hanyuda M Negishi M Ide H Adachi M Vascular endothelial growth factor expression and regulation of murine collagen-induced arthritis J Immunol 2000 15 5922 5927 10820274 10.4049/jimmunol.164.11.5922

[B25] Malik NM Jin P Raatz Y Sumariwalla PF Kiriakidis S Shepard M Feldmann M Paleolog EM Regulation of the angiopoietin-Tie ligand-receptor system with a novel splice variant of Tie1 reduces the severity of murine arthritis Rheumatology (Oxford) 2010 15 1828 1839 10.1093/rheumatology/keq163 20547659

[B26] Luttun A Tjwa M Moons L Wu Y Angelillo-Scherrer A Liao F Nagy JA Hooper A Priller J De Klerck B Compernolle V Daci E Bohlen P Dewerchin M Herbert JM Fava R Matthys P Carmeliet G Collen D Dvorak HF Hicklin DJ Carmeliet P Revascularization of ischemic tissues by PlGF treatment, and inhibition of tumor angiogenesis, arthritis and atherosclerosis by anti-Flt1 Nat Med 2002 15 831 840 12091877 10.1038/nm731

[B27] Yoo SA Joon HJ Kim HS Chae CB De Falco S Cho CS Kim WU Role of placenta growth factor and its receptor flt-1 in rheumatoid inflammation: a link between angiogenesis and inflammation Arthritis Rheum 2009 15 345 354 10.1002/art.24289 19180491

[B28] Fiedler U Reiss Y Scharpfenecker M Grunow V Koidl S Thurston G Gale NW Witzenrath M Rosseau S Suttorp N Sobke A Herrmann M Preissner KT Vajkoczy P Augustin HG Angiopoietin-2 sensitizes endothelial cells to TNF-α and has a crucial role in the induction of inflammation Nat Med 2006 15 235 239 10.1038/nm1351 16462802

[B29] Matsumoto Y Tanaka K Hirata G Hanada M Matsuda S Shuto T Iwamoto Y Possible involvement of the vascular endothelial growth factor-Flt-1-focal adhesion kinase pathway in chemotaxis and the cell proliferation of osteoclast precursor cells in arthritic joints J Immunol 2002 15 5824 5831 12023386 10.4049/jimmunol.168.11.5824

[B30] Suzuki T Miyamoto T Fujita N Ninomiya K Iwasaki R Toyama Y Suda T Osteoblast-specific Angiopoietin 1 overexpression increases bone mass Biochem Biophys Res Commun 2007 15 1019 1025 10.1016/j.bbrc.2007.08.099 17825261

[B31] Jeong BC Kim HJ Bae IH Lee KN Lee KY Oh WM Kim SH Kang IC Lee SE Koh GY Kim KK Koh JT COMP-Ang1, a chimeric form of Angiopoietin 1, enhances BMP2-induced osteoblast differentiation and bone formation Bone 2010 15 479 486 10.1016/j.bone.2009.09.019 19782780

[B32] Bergers G Hanahan D Modes of resistance to anti-angiogenic therapy Nat Rev Cancer 2008 15 592 603 10.1038/nrc2442 18650835 PMC2874834

[B33] Weinblatt ME Kremer JM Bankhurst AD Bulpitt KJ Fleischmann RM Fox RI Jackson CG Lange M Burge DJ A trial of etanercept, a recombinant tumor necrosis factor receptor:Fc fusion protein, in patients with rheumatoid arthritis receiving methotrexate N Engl J Med 1999 15 253 259 10.1056/NEJM199901283400401 9920948

[B34] Taylor PC Feldmann M Anti-TNF biologic agents: still the therapy of choice for rheumatoid arthritis Nat Rev Rheumatol 2009 15 578 582 10.1038/nrrheum.2009.181 19798034

[B35] Finckh A Simard JF Gabay C Guerne PA SCQM physicians Evidence for differential acquired drug resistance to anti-tumour necrosis factor agents in rheumatoid arthritis Ann Rheum Dis 2006 15 746 752 10.1136/ard.2005.045062 16339288 PMC1798167

